# Leprosy Reaction in Thai Population: A 20-Year Retrospective Study

**DOI:** 10.1155/2015/253154

**Published:** 2015-10-05

**Authors:** Poonkiat Suchonwanit, Siripich Triamchaisri, Sanchawan Wittayakornrerk, Ploysyne Rattanakaemakorn

**Affiliations:** Division of Dermatology, Department of Medicine, Faculty of Medicine, Ramathibodi Hospital, Mahidol University, Bangkok 10400, Thailand

## Abstract

*Background*. Leprosy is a chronic infectious disease that presents with varying dermatological and neurological symptoms. The leprosy reactions occur over the chronic course of the disease and lead to extensive disability and morbidity. *Objective*. To analyze and identify the risk factors which contribute to leprosy reactions. *Methods*. In a retrospective study, we reviewed the medical records of leprosy patients registered at the leprosy clinic, Ramathibodi Hospital, Thailand, between March 1995 and April 2015. One hundred and eight patients were included; descriptive analysis was used for baseline characteristics and a binary logistic regression model was applied for identifying risk factors correlated with leprosy reactions. *Results*. Of the 108 cases analyzed, 51 were male and 57 were female. The mean age of presentation was 45 years. The borderline tuberculoid type was the most common clinical form. Leprosy reactions were documented in 61 cases (56.5%). The average time to reaction was 8.9 months. From multivariate analysis, risk factors for leprosy reactions were being female, positive bacillary index status, and MB treatment regimen. *Conclusions*. Leprosy reactions are common complications in leprosy patients. Being female, positive bacillary index status, and multibacillary treatment regimen are significantly associated with the reactions. Early detection in cases with risk factors followed by appropriate treatment could prevent the morbidity of leprosy patients.

## 1. Introduction

Leprosy is a chronic disease caused by* Mycobacterium leprae*, which primarily affects the skin and nerves. Clinical characteristics are anesthetic skin lesions and peripheral nerve thickening. With the success of multidrug therapy (MDT) by the World Health Organization (WHO) in 1982, attention has changed to focus on the leprosy reactions, which are now the most significant problem in the management of patients.

Leprosy reactions are acute inflammatory processes occurring over the course of the disease. The reactions affect skin and nerves resulting in physical disability of the patients. There are two types of leprosy reaction. Type 1 reaction (reversal reaction, RR) is the development of acute erythema and the swelling of existing skin lesions due to the cell-mediate immunity. Type 2 reaction (erythema nodosum leprosum, ENL) is the appearance of skin nodules due to the formation of immune complexes in the humoral immunity. Reaction episodes can take place at any time during the treatment course and can be aggravated by stress, infection, or pregnancy [1, 2].

Physical disabilities caused by leprosy reactions result from nerve damage during immunological processes. The high frequency of neuritis leads to significant morbidity. The most important strategies to prevent disability are early diagnosis and treatment of both leprosy and its reactions and the provision of education to the patients [3]. To be successful in this issue, it is important to discover the risks of developing leprosy reactions.

Despite a large number of leprosy cases, publication of data concerning leprosy reactions has been limited. The information about reactions in Thailand is incomplete and scant. The purpose of this study is to identify the risk factors of leprosy reactions and to provide the epidemiological data and clinical characteristics of the patients presenting to the leprosy clinic, Ramathibodi Hospital, Thailand, between March 1995 and April 2015.

## 2. Material and Methods

This was a retrospective study conducted at the Division of Dermatology, Ramathibodi Hospital, Mahidol University, Bangkok, Thailand. The study was approved by the Mahidol University Institutional Review Board for Ethics in Human Research and was conducted in accordance with the Declaration of Helsinki.

We reviewed the medical records of leprosy patients registered at the leprosy clinic of Ramathibodi Hospital between March 1995 and April 2015 and excluded those of incomplete medical records. Clinical and demographic data were collected from the records and evaluated by simple descriptive analysis.

In the present study, treatment regimens followed the modified WHO-recommended MDT. Paucibacillary (PB) patients received rifampicin and dapsone for 6 months, while multibacillary (MB) patients were treated with rifampicin, dapsone, and clofazimine for 24 months. Release from treatment (RFT) period was the follow-up period after completion of MDT up to 5 years. Late RR was defined as a development of RR after 6 months of MDT completion. Recurrent reaction was defined as recurrence of leprosy reaction (RR or ENL) more than 6 weeks after completing the treatment of previous reaction.

The statistical analysis was performed using IBM SPSS Statistics version 21.0. Descriptive analysis was used for the baseline characteristics. Univariate and multivariate analyses of factors correlated with leprosy reaction were performed using the binary logistic regression model. Variables with a *p* value less than or equal to 0.2 in the univariate analysis were included in the multivariate analysis. Correlations were expressed as an odds ratio (OR) and as a 95% confidence interval. A *p* value less than 0.05 was considered to be statistically significant.

## 3. Results

Of the 137 cases in the leprosy clinic, 29 patients were excluded because of incomplete medical records and loss of follow-up. One hundred and eight patients were eligible for this study.

The demographic and clinical characteristics of the patients were summarized in Table 1. The patients were 57 females (52.8%) and 51 males (47.2%). The range of ages was 21 to 71 years with a mean age of 45. Fifty-nine patients lived in Bangkok, the capital city, and 7 patients had a history of leprosy contact. Borderline tuberculoid (BT) was the most common clinical form of leprosy, followed by borderline lepromatous (BL), lepromatous (LL), tuberculoid (TT), indeterminate (I), and borderline borderline (BB) forms, respectively. The bacillary index (BI) value was positive in 59 patients with a mean BI value of 3.4. According to the types of MDT received, the MB cases were 53.7%, while the PB cases were 46.3%.

**Figure 1:**
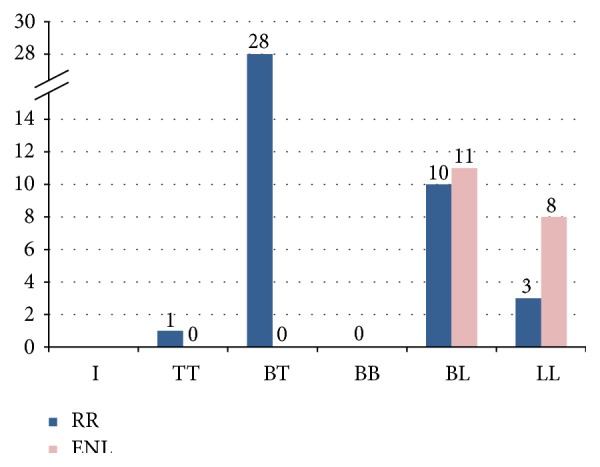
Distribution of the patients with leprosy reactions according to the clinical form.

The characteristics of leprosy reactions in our study were shown in Table 2. The reactions were documented in 61 cases (56.5%) and were predominant in the female patients. The average time to develop leprosy reaction was 8.9 months after starting treatment. Most reactions occurred in more than 6 to 12 months, during MDT treatment (34.4%). Type 1 reaction (RR) occurred more often than type 2 (ENL). BT leprosy was the form that most frequently developed leprosy reactions (Figure 1). Of the 42 patients with RR, 28 patients had evidence of skin reactions only, and the remaining 14 had both skin and nerve involvement, whereas in patients having ENL 11 had cutaneous involvement and the remaining 8 had skin and nerve involvement. Thirteen patients were diagnosed with reactions on the first visit, before the MDT treatment. Late reversal reactions were observed in 4 patients (6.6%). Nineteen patients (31.2%) experienced recurrent leprosy reactions which continued for up to 2 years.

For the univariate analysis, BI status and treatment regimen were significant risk factors for leprosy reactions (both had *p* value = 0.01). The following variables were included in the multivariate analysis (Table 3): female gender, history of leprosy contact, clinical presentation of BL and LL, positive BI status, and MB treatment regimen. When using multivariate analysis, female gender, positive BI status at diagnosis, and MB treatment regimen were risk factors for development of leprosy reactions (*p* value = 0.014, 0.004, and 0.012, resp.) (Table 4).

## 4. Discussion

Leprosy reactions are serious complications among leprosy patients. The reactions cause permanent nerve damage, resulting in disability and deformities. To prevent this morbidity, it is important to discover the risk of developing leprosy reactions. This study reported the epidemiological data, clinical characteristics, and risk factors to develop leprosy reactions in the patients registered at the leprosy clinic, Ramathibodi Hospital, Thailand, between March 1995 and April 2015.

The transmission of leprosy is person to person and depends on individual immunological conditions [4, 5]. Interestingly, our study reported that only six patients had a history of leprosy contact. The possible explanation could be disease transmission from patients with subclinical or unrecognized disease. For the type of leprosy, BT was the most common clinical form found in our study. This was consistent with the previous study in Thailand [6]. Our study reported that BB had the lowest prevalence. It is due to the fact that BB is the most unstable form of leprosy [7].

The frequency of leprosy reactions has been studied by several authors. In the present study, leprosy reactions occurred in 61 patients (56.5%). Most of the patients developed the reactions during the treatment and reactions were more prevalent in the patients treated with the MB regimen. The reactions in our study were predominantly in the RR category (68.8%). This is in contrast to the study from Thailand in 1994, when Scollard et al. reported that ENL was more prevalent than RR [8, 9]. ENL reactions were reported to occur in more than 50% of leprosy cases in the pre-MDT era [10]. The incidence of ENL reactions appears to have fallen with the use of the MDT regimen because of the combination of the bactericidal effect and the anti-inflammatory effect in MDT [11]. The varied frequencies of ENL could be due to the subjects in the studies, patients in the field, and patients reporting to the hospitals. Due to the use of widely different case definitions, it is difficult to compare the frequencies of leprosy reactions from different centers. Other factors contributing to the variation could be the duration of MDT, duration of the steroid regimen, and quality of the local leprosy control program.

Leprosy reactions were commonly found during the MDT and sometimes after release from treatment. Our study revealed that the reactions frequently developed in more than 6 to 12 months, during MDT. The average time to reaction was 8.9 months. The finding is in agreement with previous studies that leprosy reactions occur most frequently within 6 to 12 months after starting treatment [12]. The mechanism of leprosy reaction developing after treatment could be the intense release of microbial antigens due to the antibacterial action of MDT. However, most previous studies, including the present study, did not report a long term follow-up period. Because the leprosy reactions can also be observed at the first diagnostic visit, this shows that the reactions are not necessarily only the result of the treatment. In our study, 13 patients were diagnosed with reactions at the time of the first visit. These findings are in agreement with previous studies from India [13]. This may be explained by the genetic susceptibility regarding the previous reports [14, 15].

Regarding the clinical form of leprosy, our study reported greater prevalence in the group classified as lepromatous end (BL and LL). This association could be explained by the fact that there were more cases in the study, and it related immunological response to the infection due to bacterial load and higher exposure to organisms.

In leprosy reactions, the involvement of the skin and nerves occurred either singly or together. Our study found that cutaneous involvement developed more often than involvement of both skin and nerves. The observations emphasize that neuritis can occur along with inflammatory skin lesions or independently. Examination of nerves should be performed on each visit to detect early signs of nerve inflammation, regardless of the presence or absence of any skin reaction.

Late reversal reaction occurs mostly within the first 3 to 4 years after completing the treatment [16, 17]. In our study, late RR developed in 4 patients (6.6%) and the time taken to develop that ranged between 8 months and 3 years after treatment. In the absence of a clear definition for late RR and because of the different MDT regimens used in various studies, an exact comparison of the frequencies is not possible.

Recurrent episodes of leprosy reactions are an important clinical phenomenon, which may result in continuing nerve damage and add to the degree of impairment. Our study reported that 31.2% of patients had recurrent episodes which continued for up to 2 years. To prevent the consequences, patients must be informed of the possibility and be educated to return for follow-up assessment. In addition, physicians should be aware of the need to diagnose even late reactions in the long term follow-up period.

From the multivariate analysis, risk factors for leprosy reactions identified to be significant in this study were sex, BI status, and treatment regimens. Females were at greater risk of developing leprosy reactions than males. Positive BI status patients and MB treated patients showed a higher tendency towards leprosy reactions. These results were similar to those of the study by Kumar et al. [13].

The most important limitation in this study is the small number of patients, which may therefore not allow correlation to be established with confidence. Another limitation is incomplete data due to the retrospective design of the study. Because our hospital is a tertiary referral center, the population in our study may not represent the general population. Some patients were referred with atypical symptoms or were susceptible to leprosy reactions. Finally, the follow-up time in our study was too short to evaluate the long term outcomes. To minimize the impact of these limitations, prospective long term studies using a large number of patients should be performed in the future.

In conclusion, this is the study of leprosy patients from the leprosy clinic, Ramathibodi Hospital, Thailand. Reversal reaction and erythema nodosum leprosum are common complications in leprosy patients. Female gender, a positive bacteriological index, and an MB treatment regimen were found to be the main risk factors for the occurrence of leprosy reactions. Although leprosy was expected to be eliminated by 2005, those patients who have successfully completed treatment still continue to develop late or recurrent leprosy reactions. Early detection in cases where risk factors exist and prompt treatment could prevent the disabilities that cause suffering to leprosy patients and their families.

## Figures and Tables

**Table 1:** Epidemiological characteristics and background clinical status.

	Number	Percentage
Age range	21–71 (mean = 45 years)	
Gender		
Male	51	47.2%
Female	57	52.8%
Residence		
Bangkok	59	54.6%
Other areas	49	45.4%
History of leprosy contact		
Yes	7	6.5%
No	101	94.5%
Clinical form of leprosy		
TT	19	17.6%
BT	35	32.4%
BB	2	1.9%
BL	28	25.9%
LL	20	18.5%
I	4	3.7%
BI status at diagnosis		
Negative	49	45.4%
Positive	59	54.6%
Treatment regimen		
PB	50	46.3%
MB	58	53.7%
Leprosy reaction		
No reaction	47	43.5%
Yes	61	56.5%
Reversal reaction	42	38.9%
ENL	19	17.6%

Total 108 patients		

**Table 2:** Characteristics of leprosy reactions.

	Number	Percentage
Average time to develop reaction	8.9 months	
Onset of reaction		
At first diagnosis	13	21.3%
During MDT	44	72.1%
0–6 months	12	19.7%
>6–12 months	21	34.4%
>12 months	11	18%
After release from treatment	4	6.6%
0–6 months	—	
>6–12 months	3	5%
>12 months	1	1.6%
Gender		
Female	37	60.6%
Male	24	39.4%
Leprosy reaction		
Reversal reaction	42	68.8%
ENL	19	31.2%
Organ involvement in reaction		
Cutaneous	39	63.9%
Reversal reaction	28	45.9%
ENL	11	18%
Cutaneous and neuritis	22	36.1%
Reversal reaction	14	22.9%
ENL	8	13.2%
Recurrent reaction		
Yes	19	31.2%
No	42	68.8%

Total 61 patients		

**Table 3:** Univariate analysis.

Variables	Reaction			Odds ratio	95% CI	*p* value
	Yes	No	*n*			
Age range						
0–35	34	24	58	1.21	0.56–2.58	0.62
>35	27	23	50			
Gender						
Female	37	20	57	2.08	0.96–4.51	0.06^*∗*^
Male	24	27	51			
Residence						
Bangkok	31	28	59	0.7	0.32–1.51	0.36
Other areas	30	19	49			
History of leprosy contact						
Yes	6	1	7	5.02	0.58–43.21	0.10^*∗*^
No	55	46	101			
Clinical form of leprosy						
Tuberculoid (TT and BT)	29	25	54			
Lepromatous (BL and LL)	32	16	48	1.72	0.77–3.85	0.20^*∗*^
I and BB	0	6	6			
BI status at diagnosis						
Negative	28	21	49			
Positive	33	26	59	2.65	1.24–5.65	0.01^*∗*^
Treatment regimen						
PB	22	28	50			
MB	39	19	58	2.61	1.19–5.71	0.01^*∗*^

^*∗*^Including multivariate analysis.

**Table 4:** Multivariate analysis.

Variables	Odds ratio	95% CI	*p* value
Female gender	1.87	1.05–3.31	0.014^*∗*^
History of leprosy contact	3.69	0.67–14.21	1.00
Lepromatous (BL and LL)	1.2	0.84–1.35	0.84
Positive BI status	1.75	1.19–2.56	0.004^*∗*^
MB treatment regimen	1.45	1.06–4.21	0.012^*∗*^

^*∗*^Statistically significant.
